# ﻿Revision of the genus Digonis (Lepidoptera, Geometridae): new species and new genera

**DOI:** 10.3897/zookeys.1216.129923

**Published:** 2024-10-24

**Authors:** Mario I. Ramos-González, María Francisca Venegas-González, Carlos Zamora-Manzur, Luis E. Parra

**Affiliations:** 1 Departamento de Zoología, Facultad de Ciencias Naturales y Oceanográficas, Universidad de Concepción, Casilla 160-C, Concepción, Chile; 2 Programa de Doctorado en Sistemática y Biodiversidad, Facultad de Ciencias Naturales y Oceanográficas, Universidad de Concepción, Concepción, Chile; 3 Departamento de Ecología, Facultad de Ciencias, Universidad Católica de la Santísima Concepción, Alonso de Rivera 2850, Concepción, Chile

**Keywords:** Andean Region, Argentina, Chile, Ennominae, Ennomini, Nacophorini, taxonomy

## Abstract

The taxonomic study of the Chilean Ennomini genera is still in its early stages. Within this group, the maculation patterns of Chilean species are uniform and often inadequate for distinguishing between many species, compounded by a lack of taxonomic revisions focused on the genera within the tribe. In this study, the genus *Digonis* Butler, 1882, is reviewed and redefined based on characteristics of wing patterns and genitalia. *Digonis* comprises the following five species: *D.aspersa* Butler, 1882, *D.cervinaria* (Blanchard, 1852), *D.punctifera* Butler, 1882, *D.gungnir* Ramos-González & Parra, **sp. nov**., and *D.apocrypha* Ramos-González & Parra, **sp. nov.** Additionally, *D.cuprea* Butler, 1882 is synonymized with *D.cervinaria* (Blanchard, 1852), and all varieties of *D.punctifera* Butler, 1882, and *D.cuprea* are synonymized with their respective species. Furthermore, two new genera are introduced: *Phasmadigonis* Ramos-González & Parra, **gen. nov.**, erected for *P.alba* (Butler, 1882), **comb. nov.**, and *Gugnelve* Ramos-González & Parra, **gen. nov.**, established for *G.butleri* Ramos-González & Parra, **sp. nov.** With the species and genus descriptions, a comparative diagnosis, genitalia illustrations for all species, and wing venation for each genus are provided.

## ﻿Introduction

Ennominae is the most diversified subfamily, comprising approximately half of the described species within Geometridae (Gaston et al. 1995; Scoble 1999; Pitkin 2002; Scoble and Hausmann 2007; Rajaei et al. 2022). However, due to this extensive diversity (approximately 10,000 species), the taxonomic classification at the tribal level has remained in constant flux, and there is still no consensus on the taxonomic affinities of the recognized tribes, particularly when considering taxa from the New World (Holloway 1993; Scoble 1995; Pitkin 2002; Beljaev 2006; Õunap et al. 2011; Brehm et al. 2019).

The most diverse tribes within the Chilean Ennominae, which are also better studied, correspond to Odontoperini with almost 50 species (Rindge 1973, 1983; Parra and Henriquez-Rodriguez 1993; Pitkin 2002; Brehm et al. 2019), and Diptychini with approximately 40 species (Rindge 1986; Parra 1999a, 1999b; Pitkin 2002; Parra et al. 2010; Parra and Hernández 2010; Brehm et al. 2019). In contrast, there have been few taxonomic studies conducted on the other tribes (e.g., Gnophini: Parra and Vargas 2000; Macariini: Vargas et al. 2005, 2020; Boarmiini: Vargas 2007, 2021; Nacophorini: Rindge 1973, 1983; Parra 2018; Ennomini: Bocaz et al. 2016). Therefore, there is a great uncertainty regarding the monophyly of many genera and their position in the systematics of the group (Hausmann and Parra 2009; Brehm et al. 2019).

The tribe Ennomini is characterized by having a vinculum divided ventrally by a membranous area, a paired m3 muscle inserted distally in the median invagination in the basal portion of the juxta. Among Chilean genera of Ennomini, there is notable variability in wing maculation patterns, which has posed challenges in correctly recognizing species. This is exemplified in genera such as *Syncirsodes* Butler, 1882 (Bocaz and Parra 2005; Bocaz et al. 2016), *Hasodima* Butler, 1882 (Parra and Pascual-Toca 2003; Parra et al. 2009), *Perusia* Herrich-Schäffer, 1855 (CZ-M, unpublished data), and *Digonis* Butler, 1882.

*Digonis* was described by Butler (1882) based on the wing shape and the simple antennae of adults. The forewings exhibit a very acute apex, followed by a concave outer margin that extends to vein M_3_, where it terminates in a new acute angle, giving the wings the appearance of having two pointed extensions along their outer margin (Pitkin 2002).

Scoble (1999) recognized five species within *Digonis*: four originally described by Butler (1882): *D.aspersa*, *D.alba*, *D.punctifera*, including the varieties *maculosa*, *acuminata*, *terranea*, and *fumosa*, and *D.cuprea*, including the varieties *olivacea* and *fusca*. Additionally, Scoble (1999) included one species, *D.cervinaria* (Blanchard, 1852). Subsequently, in his review of neotropical Ennominae genera, Pitkin (2002) identified the species comprising the genus as *D.aspersa*, *D.cervinaria*, *D.cuprea*, and *D.punctifera*, excluding *D.alba* due to significant differences in male genitalia.

Despite the general characterization of the genus provided by Pitkin (2002), uncertainty still needs to be resolved surrounding the validity of the described species and varieties. The maculation patterns are deceptively uniform and insufficient for species recognition (e.g., Hausmann and Parra 2009). Therefore, the aim of this contribution is to characterize the genus *Digonis* through a taxonomic revision based on external morphology and genital structures. To achieve this, we redescribe the genus *Digonis*, presenting its diagnostic characters; we provide an illustrated and annotated systematic species list, along with new taxonomic changes, and describe two new genera and three new species.

## ﻿Materials and methods

This study was based on 107 adult specimens deposited in the collections mentioned below. The type material examined for each taxon is specified in the Results section.

**NHMUK**The Natural History Museum, London (UK)

**MNHN**Muséum National d’Histoire Naturelle, Paris (France)

**MNNC**Museo Nacional de Historia Natural, Santiago (Chile)

**MHNC**Museo de Historia Natural de Concepción, Concepción (Chile)

**MZUC-UCCC**Museo de Zoología de la Universidad de Concepción, Concepción (Chile)

**ZSM**Zoologische Staatssammlung München, Munich (Germany)

For specimen identification and comparison, we used the original descriptions and type material when available. Adult specimens were externally photographed using a Sony Cybershot DSC-HX300 compact camera. Wing and genitalia slides were prepared following the methods outlined in Parra (1991). Nomenclature for genitalia and external characteristics followed Klots (1970) and Scoble (1995), respectively. All prepared slides were photographed using a Motic SMZ-171-TL trinocular stereoscopic microscope equipped with a 5-Mpx Moticam Motic2500 digital camera. These photographs were then used to create detailed illustrations of the microscopic preparations.

Information on the flight period and species distribution was obtained from the labels accompanying each examined specimen. This information was supplemented with records from the citizen science platform iNaturalist (https://www.inaturalist.org). All iNaturalist records cited were identified by the first author as part of the “Polillas de Chile” project (https://inaturalist.mma.gob.cl/projects/polillas-de-chile).

All species were assigned to the biogeographical provinces proposed by Morrone (2015). The newly described genera were diagnosed through comparison with the type species of other closely related genera in the region, based on both external and internal morphological similarities.

## ﻿Results

### ﻿Taxonomy

**Tribe Ennomini Duponchel, 1845**

#### ﻿ Digonis

Taxon classificationAnimaliaLepidopteraGeometridae

Butler, 1882

CA0334D5-BB8C-5FC2-A58A-867F4501E35E

Digonis Butler, 1882: 360; Bartlett-Calvert 1886: 333; Angulo and Casanueva 1981: 12; Scoble 1999: 229; Pitkin 2002: 248.

##### Type species.

*Digonisaspersa* Butler, 1882. By original designation.

##### Diagnosis.

*Digonis* resembles *Digonodes* Warren, 1895, *Gonogala* Butler, 1882, and the newly proposed genus *Phasmadigonis* gen. nov. particularly due to the mucronate shape of the wings. However, it is distinguished from *Digonodes* by the presence of a mucronate outer margin on the M_3_ of the hindwings and the presence of filiform (not bipectinate) antennae. It is recognized as distinct from *Gonogala* by the absence of bipectinate antennae and M_2_ in the hindwings. It differs from *Phasmadigonis* by the presence of two accessory areoles in the forewings, R_2_ arising from R_3+4_, and the absence of a vein connecting Sc+R_1_ to the discal cell in the hindwings. The monophyly of *Digonis* is supported by the following genital characters: a U-shaped gnathos with a plate or a pair of denticulate lobes, a concave, sclerotized process on the costa with an extended cucullus, a furca armed with spines, an aedeagus without cornuti, and a strongly denticulate annular signum.

##### Redescription.

**Figure 1.**
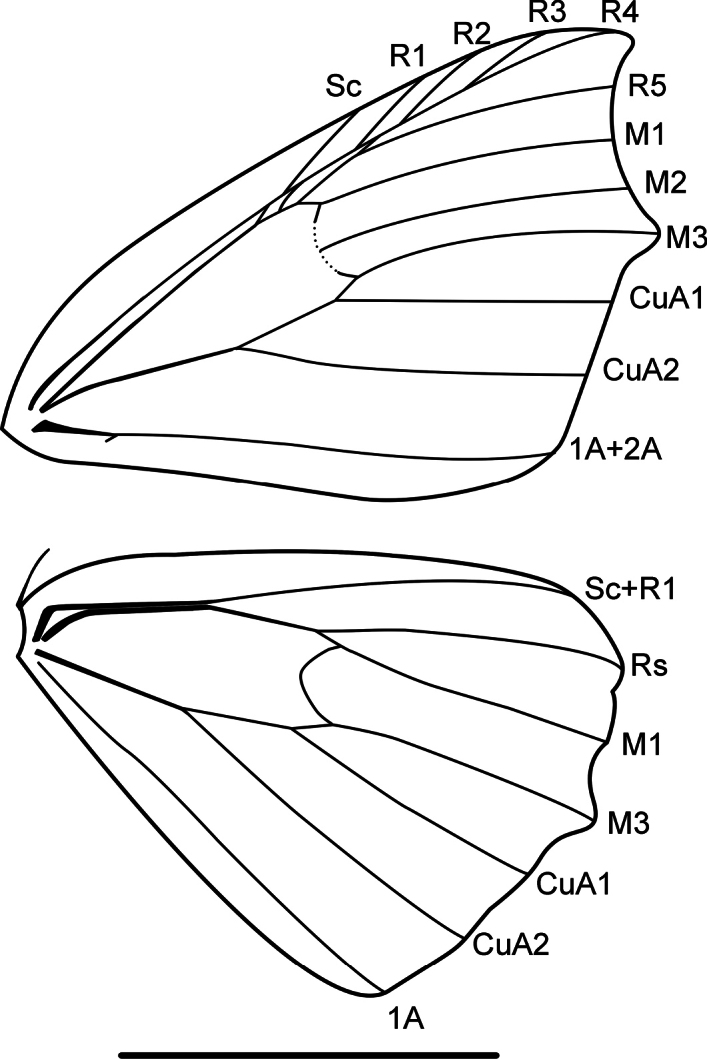
Wing venation of *Digonis* Butler, 1882. Scale bar: 10 mm.

Antennae serrate in males and filiform in females. Thorax and abdomen with brown to grayish scales. Forewings castaneous yellowish, gray, brown, or coppery; costal margin in the apical area slightly arched; outer margin concave between apex and M_3_ vein. Wing venation (Fig. 1). Two accessory cells; Sc in contact with first accessory cell, R_1_ originates near apex of second accessory cell, R_2+3+4_ from apex of second accessory cell, R_3_ and R_4_ stalked, R_5_ terminates at termen; M_2_ equidistant between M_1_ and M_3_, M_3_ slightly arched and ending in small mucronate apex; CuA_1_ originates 1/10 before end of cell, CuA_2_ originates near the midpoint of the cell. Hindwings paler than forewings, with multiple dark brown scattered spots; medial band conspicuous or faint; outer margin slightly mucronate. Sc+R_1_ in contact with radial stem up to middle of cell, Rs originates 1/10 before end of cell, M_2_ absent. Male genitalia with conical uncus; gnathos “U” shaped, with a pair of prominences or a denticulated plate; subrectangular valvae with a strong sclerotized and concave costal process; spiny furca; aedeagus unarmed. Female genitalia with subpyriform corpus bursae, annular and strongly denticulated signum.

##### Distribution.

This genus is distributed between latitudes 30°S and 47°S, spanning the provinces from Elqui to Capitán Prat in Chile.

#### ﻿ Digonis aspersa

Taxon classificationAnimaliaLepidopteraGeometridae

Butler, 1882

A5A4C7AF-1AD6-59D3-B6B9-D831F1206E62

Figs 2A–D , 3

Digonis aspersa Butler, 1882: 361; Bartlett-Calvert 1886: 333; Angulo and Casanueva 1981: 12; Scoble 1999: 229; Pitkin 2002: 248.

##### Material examined.

***Syntype*.** Chile • 1 male; Pines Valley; “XII” [labeled]; “Chili, 82-107” [labeled]; “Type” [red labeled]; T. Edmonds leg.; NHMUK.

##### Other material examined.

Chile — **San Antonio Prov.** • Algarrobo; 10-I-1950; n.n. leg.; MZUC-UCCC. — **Diguillín Prov.** • 1 male; Ninhue; 19-IV-2011; G. Moreno leg.; MZUC-UCCC • Las Trancas; III-2013; -36.889694, -71.471611; “Mirg-006” [genitalia slide]; MZUC-UCCC • Las Trancas; 16-IV-2010; G. Moreno leg.; “UCCC-MZUC-Lep 0258” [Museum ID]; MZUC-UCCC • 1 female; Las Trancas; 8-II-2011; G. Moreno leg.; “UCCC-MUZ-Lep-1056” [Museum ID]; “Mirg-022” [genitalia slide]; MZUC-UCCC • Las Trancas; 20-I-2012; G. Moreno leg.; “UCCC-MZUC-Lep 0377” [Museum ID]; MZUC-UCCC. — **Concepción Prov** • 2 males; Concepción; 8-I-2001; J. Artigas leg.; MZUC-UCCC • 1 male; Concepción, Cerro Caracol; 15-II-2001; J. Artigas leg.; “Mirg-003” [genitalia slide]; MZUC-UCCC • 1 male; Concepción, Cerro Caracol; 30-II-2002; J. Artigas leg.; MZUC-UCCC • 1 male; Concepción; 3-II-1960; Trampas leg.; MZUC-UCCC • Concepción; 22-I-1960; Trampas leg.M MZUC-UCCC • Concepción; 1-IV-1960; Trampas leg.; MZUC-UCCC • Concepción; 5-III-1959; Trampas leg.; MZUC-UCCC • Concepción; 21-III-1962; Trampas leg.; MZUC-UCCC • Concepción; 30-IX-2001; J. Artigas leg.; MZUC-UCCC • Concepción, Cerro Caracol; 30-II-2002; J. Artigas leg.; MZUC-UCCC • Concepción; 7-I-1961; Trampas leg.; MZUC-UCCC • 3 males; Concepción, Barrio Universitario; 4-V-1985; Carrasco leg.; MZUC-UCCC • Concepción; 29-XII-1958; Trampas leg.; MZUC-UCCC • Concepción; 16-III-1960; Trampas leg.; MZUC-UCCC. —**Arauco Prov.** • 1 male; Lanalhue; 22-I-2018; L. Parra leg.; “Mirg-001” [genitalia slide]; MZUC-UCCC • Lanalhue; 23-I-2018; L. Parra leg.; MZUC-UCCC. — **Biobío Prov.** • P.N. Laguna del Laja; 6-XII-2008; G. Moreno leg.; MZUC-UCCC. — **Malleco Prov.** • Nahuelbuta; Río Picoyquen; 22-XII-1960; Fetis leg.; MZUC-UCCC • Collipulli; 24-X-2014; E. Sepulveda leg.; “UCCC-MZUC-Lep 0308” [Museum ID]; MZUC-UCCC • Río Blanco; 19/25-II-1995; H. Thöny leg.; ZSM. — **Valdivia Prov.** 1 male; Valdivia; 30-IX-1987; Trampas leg.; MZUC-UCCC • 1 male; Valdivia; “5003” [Museum ID]; MNNC. — **Llanquihue Prov.** • Katalapi; 22-IV-2011; MZUC-UCCC. — **Coyhaique Prov.** • Coyhaique; I-1934; E. Ureta leg.; “Museo 5004” [Museum ID]; MNNC • 1 female; Lago Verde, R.N. Coyaique; 21-I-2007; MZUC-UCCC. — **Aysen Prov.** • 1 female; Río Maca, Cuenca Cuervo; -45.114703, -73.016065; 21-II-2008; “Mirg-020” [genitalia slide]; MZUC-UCCC. — **Capitán Prat Prov.** • Los Mellizos; 22-I-2008; Muñoz-Escobar leg.; MZUC-UCCC.

##### Additional records.

Chile. — **Itata Prov.** • La Palma; -36.567368, -72.689177; 9-IX-2021; observed by Claudio Maureira and submitted to iNaturalist in: https://inaturalist.mma.gob.cl/observations/100908464. — **Concepción Prov.** • Concepción; 4-XII-2021; -36.834994, -73.011375; observed by Flor Susana and submitted to iNaturalist in: https://inaturalist.mma.gob.cl/observations/102608301. • Concepción; 5-V-2023; -36.8354793349, -73.0287251249; observed by Luis Chavarriga and submitted to iNaturalist in: https://inaturalist.mma.gob.cl/observations/159871318. • Coliumo; 9-II-2023; -36.5526014, -72.9574049; observed by fpizarro and submitted to iNaturalist in: https://inaturalist.mma.gob.cl/observations/148464516. • Concepción; 20-XII-2023; -36.8263187829, -73.0265376168; observed by Antonio Maureira Navarrete and submitted to iNaturalist in: https://inaturalist.mma.gob.cl/observations/144894518 • Concepción; 15-XII-2022; -36.7784506232, -73.0294558602; observed by Antonio Maureira Navarrete and submitted to iNaturalist in: https://inaturalist.mma.gob.cl/observations/144446815. • Concepción; 20-XII-2023; -36.8263187829, -73.0265376168; observed by Antonio Maureira Navarrete and submitted to iNaturalist in: https://inaturalist.mma.gob.cl/observations/144894518 — **Biobío Prov.** • Polcura; -37.285095, -71.718547; 20-III-2022; observed by Flor Susana and submitted to iNaturalist in: https://inaturalist.mma.gob.cl/observations/110011024. — **Osorno Prov.** • Osorno; -40.565175, -73.161816; 19-VI-2021; observed by Ricardo Huenuanca and submitted to iNaturalist in: https://inaturalist.mma.gob.cl/observations/83672367 • Osorno; -40.565183, -73.161729; 02-V-2021; observed by Ricardo Huenuanca and submitted to iNaturalist in: https://inaturalist.mma.gob.cl/observations/76765927 • Osorno; -40.565303, -73.162031; 09-I-2021; observed by Ricardo Huenuanca and submitted to iNaturalist in: https://inaturalist.mma.gob.cl/observations/67836032.

##### Diagnosis.

This species can be easily distinguished from *D.punctifera* (Butler), *D.gungnir* Ramos-González & Parra, sp. nov., and *D.cervinaria* by its brownish forewings crossed by slightly sinuous bands and bicolored spots in the postmedial band at the level of veins R_3_, R_4_, R_5_, M_1_, M_2_, M_3_, CuA_1_, CuA_2_, and 1A+2A. Externally, it differs from *D.apocrypha* Ramos-González & Parra, sp. nov. because its bicolored spots appear on both sides of both pairs of wings with an equal proportion of white and black scales. It can be easily distinguished from its congeners by three other genitalia characters: a tongue-shaped furca that does not surpass the height of the transtilla, the presence of lateral spines in the distal half of the furca, and anterior apophyses directed towards the tergum.

##### Redescription.

**Figure 2.**
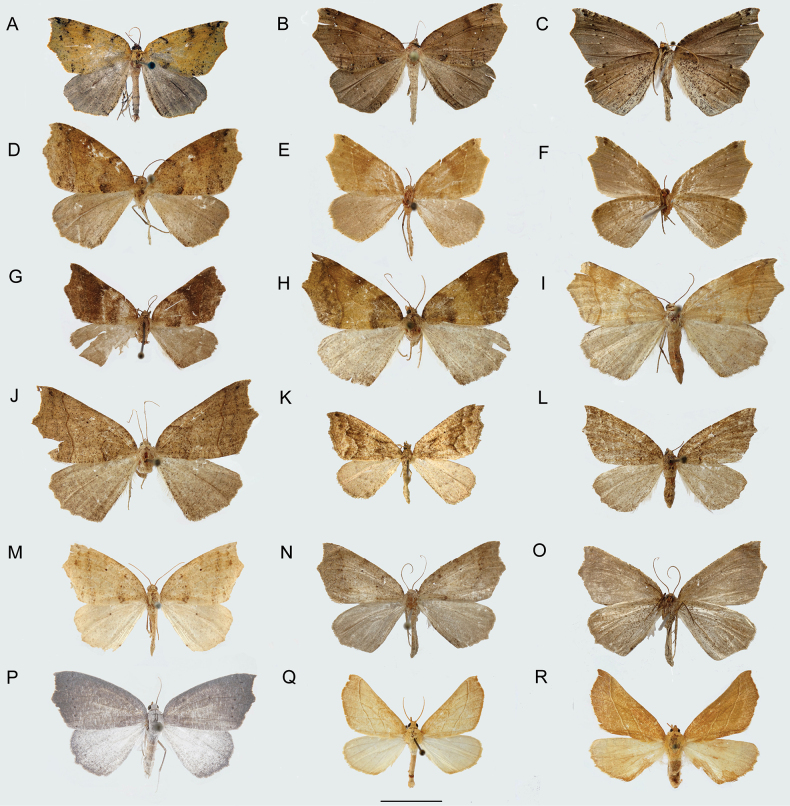
Habitus of *Digonis* adults **A***Digonisaspersa* (male in dorsal view) **B***Digonisaspersa* (male brown-morpho in dorsal view) **C***Digonisaspersa* (male brown-morpho in ventral view) **D***Digonisaspersa* (female in dorsal view) **E***Digoniscervinaria*, stat. rev. (male olivaceous-morpho in dorsal view) **F***Digoniscervinaria*, stat. rev. (male olivaceous-morpho in ventral view) **G***Digoniscervinaria*, stat. rev. (male fuscous-morpho in dorsal view) **H***Digoniscervinaria*, stat. rev. (female in dorsal view) **I***Digoniscervinaria*, stat. rev. (female olivaceous-morpho in ventral view) **J***Digoniscervinaria*, stat. rev. (female brown-morpho in ventral view) **K***Digonispunctifera* (male in dorsal view; photo courtesy of A. Hausmann) **L***Digonispunctifera* (female in dorsal view) **M***Digonisgungnir* Ramos-González & Parra, sp. nov. (holotype in dorsal view) **N***Digonisapocrypha* Ramos-González & Parra, sp. nov. (holotype in dorsal view) **O***Digonisapocrypha* Ramos-González & Parra, sp. nov. (holotype in ventral view) **P***Phasmadigonisalba*, comb. nov. (male in dorsal view) **Q***Gugnelvebutleri* Ramos-González & Parra, sp. nov. (holotype in dorsal view) **R***Gugnelvebutleri* Ramos-González & Parra, sp. nov. (allotype in dorsal view). Scale bar: 10 mm.

**Figure 3.**
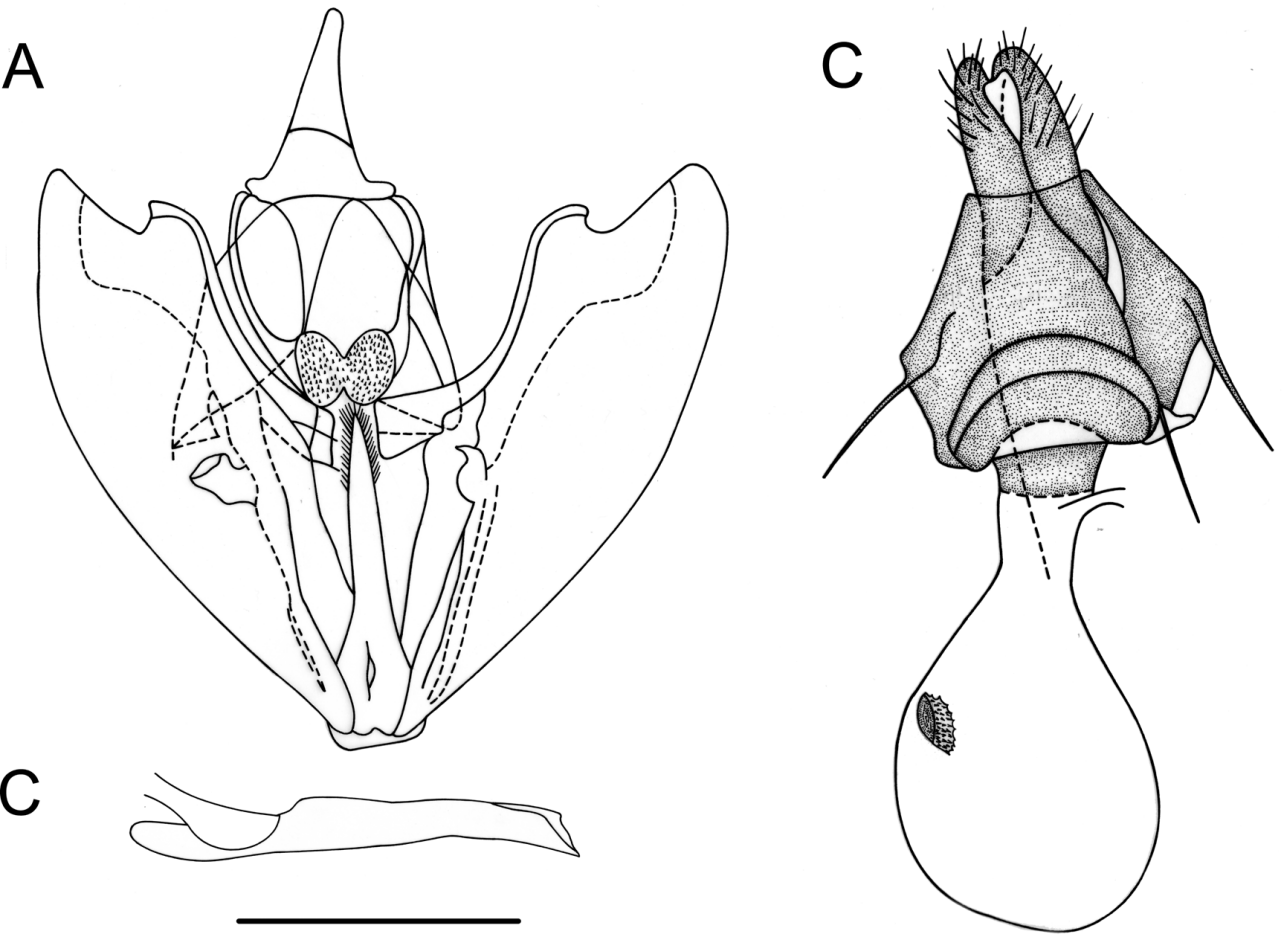
Genitalia of *Digonisaspersa***A** male genitalia in ventral view **B** aedeagus in lateral view **C** female genitalia in ventral view. Scale bar: 1 mm.

**Male** (Fig. 2A–C). Head: antennae slightly serrate; palpi long, one-third larger than eye diameter and slightly pointing upward; frons and vertex covered with mottled brownish gray scales. Thorax: patagia covered with elongated scales of same color as background; tegulae covered with piliform scales of same color as background; tibial spur formula 0-2-4. Forewings: subtriangular with acute apex and outer margin excavated between apex and M3, with a slight mucronate extension; fovea absent; background color variable, ranging from yellowish brown to dark brown with numerous small scattered blackish spots on the surface; antemedial band dark brown to blackish, slightly arched and marked by three bicolored spots (proximal half with whitish scales and distal half with blackish scales) at the level of radial, cubital, and anal veins respectively; medial band diffuse, blackish to smoky brown, more noticeable along the costal margin; postmedial band dark brown, slightly sinuous with the costal sector, strongly arched, with bicolored spots (proximal half with blackish scales and distal half with whitish scales) at the level of veins R_3_, R_4_, R_5_, M_1_, M_2_, M_3_, CuA_1_, CuA_2_, and 1A+2A, also visible on ventral side; subterminal band diffuse and marked only by dark spots in subapical region, at the level of medial veins and along the anal margin; discal spot visible, punctiform, and blackish. Hindwings: subrectangular with small mucronate apex at the level of M_3_; background color grayish brown; postmedial band slightly smoky dark brown, marked by bicolored spots (proximal half with blackish scales and distal half with whitish scales) at the level of veins, also visible on ventral surface; discal spot visible only on ventral surface. Male genitalia (Fig. 3A, B). Uncus conical, apically club-shaped; gnathos U-shaped with expanded apex forming a pair of spiny lobes; valvae subrectangular, costa strongly sclerotized with distal lobe before apex, crescent-shaped, slightly convex, and cucullus extending beyond apex of costa; transtilla truncated; saccus subrectangular; juxta emarginated anteriorly, with a median dimple, and furca straight, short, does not surpass the height of the transtilla, tongue-shaped, spiny only laterally in the distal half; anellus sclerite weak, pentagonal in shape. Aedeagus tubular, straight; vesica without cornuti.

**Female** (Fig. 2D). Similar to male, but with simple antennae and well-defined Σ-shaped antemedial band. Female genitalia (Fig. 3C). Ductus bursae one-sixth the length of corpus bursae; lamella antevaginalis wide, strongly sclerotized; corpus bursae membranous, subpyriform with an annular, hollow, strongly denticulated signum anteriorly; posterior apophyses twice as long as anterior apophyses, anterior apophyses slightly bent towards tergite.

##### Distribution.

This species is found between the Chilean provinces of San Antonio and Capitán Prat, with records in Neuquén, Argentina (Chalup 2020). It is distributed in parts of the biogeographic provinces of Santiago, Central Chilean subregion; Maule and Valdivian Forest, Subantarctic subregion, in the Andean region.

##### Flight period.

Specimens were captured or observed in January, February, March, April, May, June, September, October, and December. There are no records for other months.

##### Remarks.

Although the holotype stands out due to its yellowish tone, most examined specimens exhibit a brownish coloration. There is no evidence to suggest that this color variation reflects sexual dimorphism. The genitalia of these brown specimens are congruent with those of the holotype, indicating high variability in wing coloration.

#### ﻿ Digonis cervinaria

Taxon classificationAnimaliaLepidopteraGeometridae

(Blanchard, 1852) stat. rev.

5D40E608-E270-5E63-8F32-2B1B3173FC9B

Figs 2E–J , 4

Ennomos cervinaria Blanchard, 1852: 89.
Drepanogynis eversaria Guenée, 1858: 93 (unnecessary replacement name)
Digonis cervinaria (Blanchard, 1852); Scoble 1999: 229; Pitkin 2002: 248.
Digonis cuprea Butler, 1882: 362; Bartlett-Calvert 1886: 333; Angulo and Casanueva 1981: 12; Scoble 1999: 229; Pitkin 2002: 248. syn. nov.
Digonis cuprea fusca Butler, 1882: 362; Bartlett-Calvert 1886: 333; Angulo and Casanueva 1981: 12; Scoble 1999: 229; Pitkin 2002: 248. syn. nov.
Digonis cuprea olivacea Butler, 1882: 362; Bartlett-Calvert 1886: 333; Angulo and Casanueva 1981: 12; Scoble 1999: 229; Pitkin 2002: 248. syn. nov.

##### Material examined.

***Syntypes*** . Chile • 1 female; Coquimbo; C. Gay leg.; “*Digoniscervinaria*” [labeled]; MNHN • 1 female; Valparaíso; T. Edmonds leg.; “*Digoniscuprea*” [labeled]; “Chili, 82-107”; “Type” [labeled]; NHMUK • 2 males; same data as for preceding but “*var. fusca*” [labeled]; “Type” [labeled] “Chili, 82-107” [labeled] and “*cupreaolivacea*” [labeled]; “Type” [labeled]; “Chili, 82-107” [labeled]; all NHMUK.

##### Other material examined.

Chile — **Valparaíso Prov.** • Viña del Mar; 8-VIII-1954; “Museo 5343” [Museum ID]; MNNC) • 1 female; Viña del Mar; 18-IV-1953; “Museo 5018” [Museum ID]; “Mirg-015” [genitalia slide]; MNNC; • 1 male; Viña del Mar; 15-X-1953; “Museo 5349” [Museum ID]; MNNC • 1 male; Viña del Mar; 15-VIII-1953; “Museo 5342” [Museum ID]; MNNC • 1 female; Viña del Mar; 12-IX-1953; “Museo 5350” [Museum ID]; MNNC • 1 male; Laguna Verde; 10-X-1936; E. Ureta leg.; “Museo 5014” [Museum ID]; MNNC. — **Marga Marga Prov.** • 1 male; Poza Azul, Marga Marga; 14-XII-1953; “Museo 5346” [Museum ID]; MNNC. — **Cordillera Prov.** • 1 male; La Obra; 26-III-1953; “Museo 5373” [Museum ID]; MNNC. — **Cachapoal Prov.** • 1 female; Termas de Cauquenes; 11-I-1953; E. Ureta leg.; “Museo 5006” [Museum ID]; “Mirg-013” [genitalia slide] • 1 male; Termas de Cauquenes; 11-I-1953; E. Ureta leg.; “Museo 5023” [Museum ID]; MNNC. — **Talca Prov.** 1 female; Panguilemo, La Calor; 1-II-2005; L. Parra leg.; “Mirg-009” [genitalia slide]; MZUC-UCCC. — **Diguillín Prov.** • 1 female; Las Trancas; 29-V-2011; G. Moreno leg.; “Mirg-016” [genitalia slide]; MZUC-UCCC. — **Concepción Prov.** • 1 female; Concepción; 6-I-1961; Trampas leg.; MZUC-UCCC. — **Malleco Prov.** • 1 female; Río Blanco, Curacautín; II-1995; H. Thöny leg.; ZSM. — **Cautín Prov.** • 1 female; Termas de Río Blanco; III-1951; MZUC-UCCC. — **Valdivia Prov.** • 1 female; Valdivia; I-1974; Cameron leg.; MZUC-UCCC. — **Osorno Prov.** • 1 female; Puerto Octay; 11-III-1956; Oehrens leg.; “Museo” [labeled]; MNNC. — **Palena Prov.** • Fjord Comau, Huinay Station, -42.381111, -72.414722, 20 m; 8-I-2008; A. Hausmann leg.; ZSM.

##### Additional records.

Chile — **Itata Prov.** • La Palma; -36.567339, -72.689122; 09-VI-2021; observed by Claudio Maureira and submitted to iNaturalist in: https://inaturalist.mma.gob.cl/observations/85725251. — **Concepción Prov.** • Concepción; -36.7928173278, -73.0525010616; 13-XII-2022; observed by Antonio Maureira Navarrete and submitted to iNaturalist in: https://inaturalist.mma.gob.cl/observations/144446817. — **Cautín Prov.** • Molco; -39.3393898652, -72.0921077239; 25-XII-2022; observed by Michael Weymann and submitted to iNaturalist in: https://inaturalist.mma.gob.cl/observations/145759642. • Molco; -39.3394188914, -72.0921402457; 25-XII-2022; observed by Michael Weymann and submitted to iNaturalist in: https://inaturalist.mma.gob.cl/observations/145759626. **Osorno Prov.** • Osorno; -40.565282, -73.161883; 31-I-2021; observed by Ricardo Huenuanca and submitted to iNaturalist in: https://inaturalist.mma.gob.cl/observations/68898181 • Osorno; -40.565277, -73.162024; 3-I-2021; observed by Ricardo Huenuanca and submitted to iNaturalist in: https://inaturalist.mma.gob.cl/observations/67871022. — **Llanquihue Prov.** • Las Cascadas; -41.0799870456, -72.6350197924; 6-V-2023; observed by Mario Ramos and submitted to iNaturalist in: https://inaturalist.mma.gob.cl/observations/164957877. — **Chiloé Prov.** • Puntra; -42.119003, -73.81765; 27-II-2022; observed by Waldo Moyano and submitted to iNaturalist in: https://inaturalist.mma.gob.cl/observations/107842416.

##### Diagnosis.

This species can be distinguished externally from other *Digonis* species by having elongated whitish spots at the level of veins R3, R4, R5, M1, M2, M3, CuA1, CuA2, and 1A+2A in postmedial region, also visible on ventral view. Male genitalia are characterized by the presence of a needle-shaped furca that surpasses the height of the transtilla, with only apical spines; costa strongly sclerotized with distal lobe, expanded before apex with truncated edge, and spatulate transtilla; aedeagus with a narrow and elongated caecum, one-third of the total length of the aedeagus.

##### Redescription.

**Figure 4.**
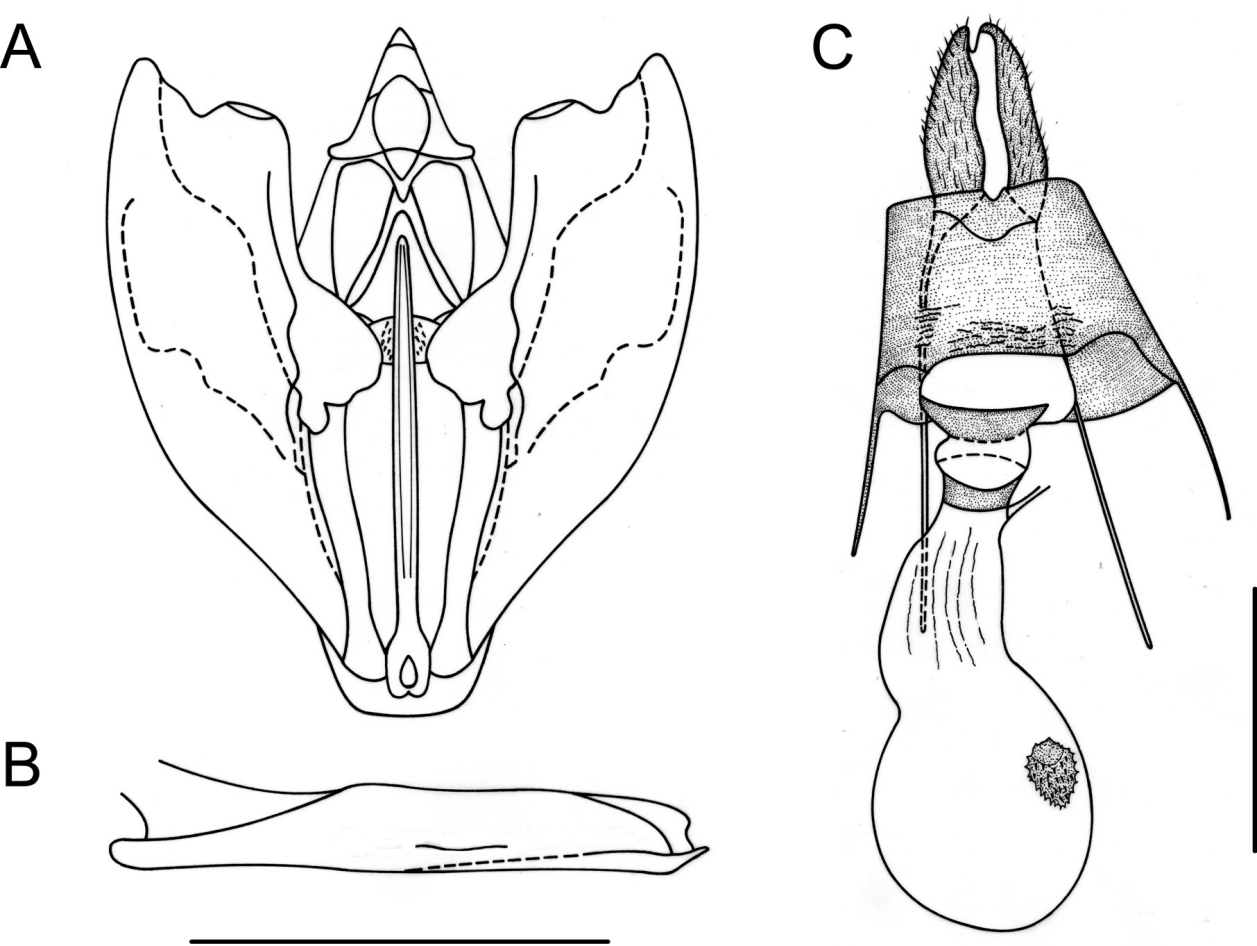
Genitalia of *Digoniscervinaria*, stat. rev. **A** male genitalia in ventral view **B** aedeagus in lateral view **C** female genitalia in ventral view. Scale bars: 1 mm.

**Male** (Fig. 2E–G). Head: antennae slightly serrate; palpi short, subequal to eye diameter, porrect; frons and vertex covered with juxtaposed brownish scales. Thorax: patagia covered with elongated scales of same color as background; tegulae covered with piliform scales of same color as background; tibial spur formula 0-2-4. Forewings: subtriangular with acute apex and outer margin excavated between apex and M_3_, with slight mucronate extension; fovea absent; background color variable, ranging from olive-brown to dark brown; antemedial band dark brown to blackish, slightly sinuous; medial band diffuse, blackish to smoky brown, slightly oblique and in contact with postmedial band near anal margin; postmedial band dark brown, slightly sinuous with the costal sector, near apex, strongly arched, and with elongated whitish spots at the level of veins R_3_, R_4_, R_5_, M_1_, M_2_, M_3_, CuA_1_, CuA_2_, and 1A+2A, also visible on ventral view; subterminal band diffuse and marked only by dark spots in subapical region; discal spot visible, punctiform, and blackish. Hindwings: subrectangular with small mucronate apex at the level of M_3_; background color grayish brown; postmedial band dark brown, slightly smoky, marked by elongated whitish spots at the level of veins, only visible on ventral surface; discal spot visible only on ventral surface. Male genitalia (Fig. 4A, B). Uncus conical, apically halberd-shaped; gnathos U-shaped with an expanded apex forming a pair of spiny lobes; valvae subrectangular, costa strongly sclerotized with a distal lobe before the apex, truncated, slightly convex, and cucullus extending beyond the apex of the costa; transtilla spatulate; saccus membranous; juxta emarginated anteriorly, with a median dimple, and furca straight, longitudinally striated, long, surpassing the height of the transtilla, needle-shaped, spiny only apically; anellus sclerite weakly defined. Aedeagus tubular, straight; caecum narrow and elongated, one-third of the total length of the aedeagus; vesica without cornuti.

**Female** (Fig. 2H–J). Similar to male, but with simple filiform antennae, Σ-shaped antemedial band, and zigzagging subterminal band. Female genitalia (Fig. 4C). Ductus bursae one-fourth the length of the corpus bursae; lamella antevaginalis, sclerotized; corpus bursae membranous, subpyriform with an annular, hollow, strongly denticulated signum anteriorly; posterior apophyses three times longer than the anterior apophyses.

##### Distribution.

This species is found between the Elqui and Palena provinces. It is distributed in parts of the biogeographic provinces of Coquimbo and Santiago, Central Chilean subregion; Maule and Valdivian Forest, Subantarctic subregion, in the Andean region.

##### Flight period.

Specimens were captured or observed in January, February, March, April, May, June, August, September, October, and December. There are no records for other months.

#### ﻿ Digonis punctifera

Taxon classificationAnimaliaLepidopteraGeometridae

Butler, 1882

24D3A13E-737E-5CDD-8B64-476C599E1EFB

Figs 2K, L , 5

Digonis punctifera Butler, 1882: 363; Bartlett-Calvert 1886: 333; Angulo and Casanueva 1981: 12; Pitkin 2002: 248.
Digonis punctifera acuminata Butler, 1882: 363; Bartlett-Calvert 1886: 333; Angulo and Casanueva 1981: 12; Pitkin 2002: 248. syn. nov.
Digonis punctifera fumosa Butler, 1882: 363; Bartlett-Calvert 1886: 333; Angulo and Casanueva 1981: 12; Pitkin 2002: 248. syn. nov.
Digonis punctifera maculosa Butler, 1882: 363; Bartlett-Calvert 1886: 333; Angulo and Casanueva 1981: 12; Pitkin 2002: 248. syn. nov.
Digonis punctifera terranea Butler, 1882: 363; Bartlett-Calvert 1886: 333; Angulo and Casanueva 1981: 12; Pitkin 2002: 248. syn. nov.

##### Material examined.

***Syntypes*.** Chile • 1 male; Valparaíso; T. Edmonds Leg; “Type” [labeled]; “Chili, 82-107” [labeled]; NHMUK • 1 female; Valparaíso; T. Edmonds leg.; “*var. acuminata*” [labeled]; “Type” [labeled]; “Chili, 82-107” [labeled]; NHMUK • 1 male; Valparaíso; T. Edmonds leg.; “var.fumosa” [labeled]; “Type” [labeled]; “Chili, 82-107” [labeled]; NHMUK • 2 females; same data as for preceding but “*var. maculosa*” [labeled]; “Type” [labeled]; “Chili, 82-107” [labeled]; all NHMUK • 1 male; same data as for preceding but “*var. terranea*” [labeled]; “Type” [labeled]; “Chili, 82-107” [labeled]; NHMUK.

##### Other material examined.

Chile — **Limarí Prov.** • Ovalle, Quebrada Seca; 28/29-XI-1997; ZSM • 1 female; Ovalle, Los Molles; 16/19-X-1994; ZSM. — Petorca Prov. • 1 male and 1 female; Cachagua, Quebrada Aguas Claras; 5-III-1997; ZSM. — **Valparaíso Prov.** • 1 female; Viña del Mar; 4-IV-1953; “Mirg-011” [genitalia slide]; MNNC. • 3 males and 1 female; Valparaíso; 10-III-2006; H. Thoeny leg.; ZSM. — **Diguillín Prov.** • 1 male; Las Trancas; 8-II-2011; G. Moreno leg.; MZUC-UCCC.

##### Additional records.

Chile. — **Limarí Prov.** • P.N. Fray Jorge; -30.658155, -71.66439; 21-VII-2016; observed by Bastian Riveros and submitted to iNaturalist in: https://inaturalist.mma.gob.cl/observations/87956647.

##### Diagnosis.

Externally, *D.punctifera* is characterized by the presence of a sinuous postmedial band that follows the shape of the wing’s outer margin. The female genitalia are distinguished by a ductus bursae that is half the length of the corpus bursae and posterior apophyses five times longer than the anterior apophyses.

##### Redescription.

**Male** (Fig. 2K). Head: antennae slightly serrate; palpi long, one-third longer than the eye diameter and slightly pointing upward; frons and vertex covered with mottled grayish brown scales. Thorax: patagia covered with elongated grayish brown and dark brown scales; tegulae covered with piliform scales of same color as patagia; tibial spur formula 0-2-4. Forewings: subtriangular with acute apex and outer margin excavated between apex and M_3_, with slight mucronate extension; fovea absent; background color variable, ranging from yellowish brown to dark brown, with numerous small blackish spots scattered on surface; antemedial band faint, brownish dark, Σ-shaped; postmedial band dark brown, sinuous following the shape of the wing’s outer margin, with a strip or whitish points toward the distal margin, not visible on ventral surface; subterminal band diffuse and marked only by dark spots in subapical region, at the level of medial veins and on anal margin; discal spot visible, punctiform, and blackish. Hindwings: subrectangular with small mucronate apex at the level of M_3_; background color grayish brown; postmedial band dark brown, zigzagging, slightly smoky, and marked by blackish spots at the level of veins, also visible on ventral surface; discal spot visible, punctiform, and blackish. Male genitalia. Unknown.

**Figure 5.**
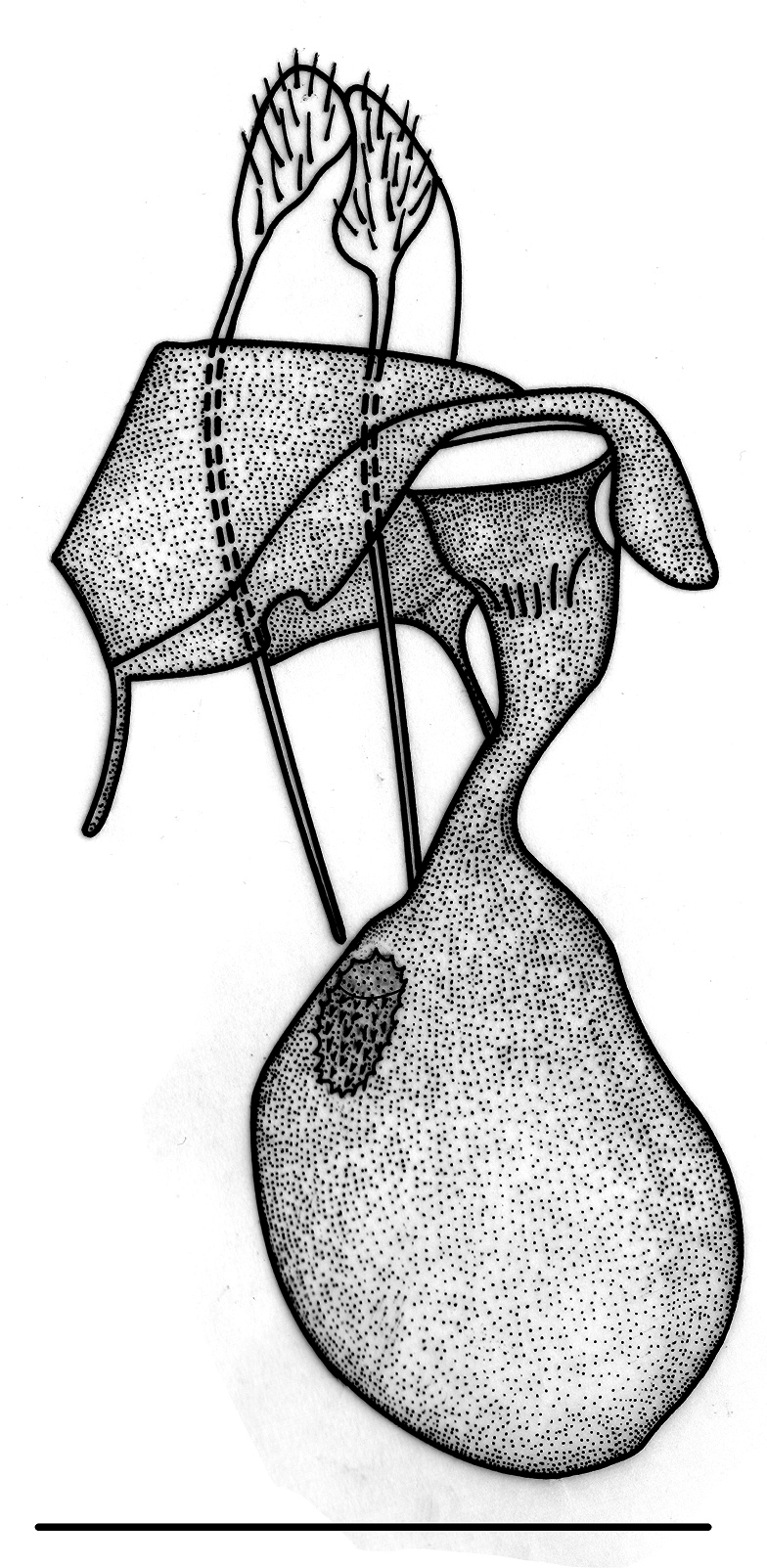
Female genitalia of *Digonispunctifera*. Scale bar: 1 mm.

**Female** (Fig. 2L). Similar to male but with simple filiform antennae and more defined antemedial band, also Σ-shaped. Female genitalia (Fig. 5). Ductus bursae half the length of the corpus bursae; lamella antevaginalis sclerotized; corpus bursae membranous, subpyriform with an annular, hollow, strongly denticulated signum anteriorly, located to the right of the corpus bursae; posterior apophyses five times longer than the anterior apophyses.

##### Distribution.

This species is found between the Limarí and Diguillín provinces. It is distributed in parts of the biogeographic provinces of Coquimbo and Santiago, Central Chilean subregion; Maule, Subantarctic subregion, Andean region.

##### Flight period.

Specimens were captured or observed in February, March, April, July, October, and November. There are no records for other months.

#### ﻿ Digonis gungnir

Taxon classificationAnimaliaLepidopteraGeometridae

Ramos-González & Parra sp. nov.

28F43E6E-E64D-5233-8716-EB62FB6B3707

https://zoobank.org/2BE6459A-9B57-400B-91F1-844437B57E79

Figs 2M , 6

##### Type material.

***Holotype*.** Chile — 1 male; pinned; Coquimbo, Elqui, Huanta; 1936 (year without more data); E. Ureta leg.; “Holotype *Digonisgungnir*” [red handwritten label]; “5024” [Museum ID]; “Mirg-019” [genitalia slide]; MNNC. ***Paratypes*.** Chile — 1 male; pinned; Llanquihue Prov., Maullín; II-1943; S. Barros leg.; MZUC-UCCC • 1 male; pinned; Magallanes Prov., Punta Arenas, Tres Puentes; XII-1952; n.n. leg.; MZUC-UCCC.

##### Diagnosis.

Externally, *D.gungnir* Ramos-González & Parra, sp. nov. is characterized by straw-colored wings and a straight postmedial band with pale points bordered in dark brown at veins R_4_, R_5_, M_1_, M_2_, and M_3_ on the forewings. Male genitalia feature a presence of a poorly defined juxta, a slightly arched spear-shaped furca, and dense dorsal spines on the furca.

##### Description.

**Figure 6.**
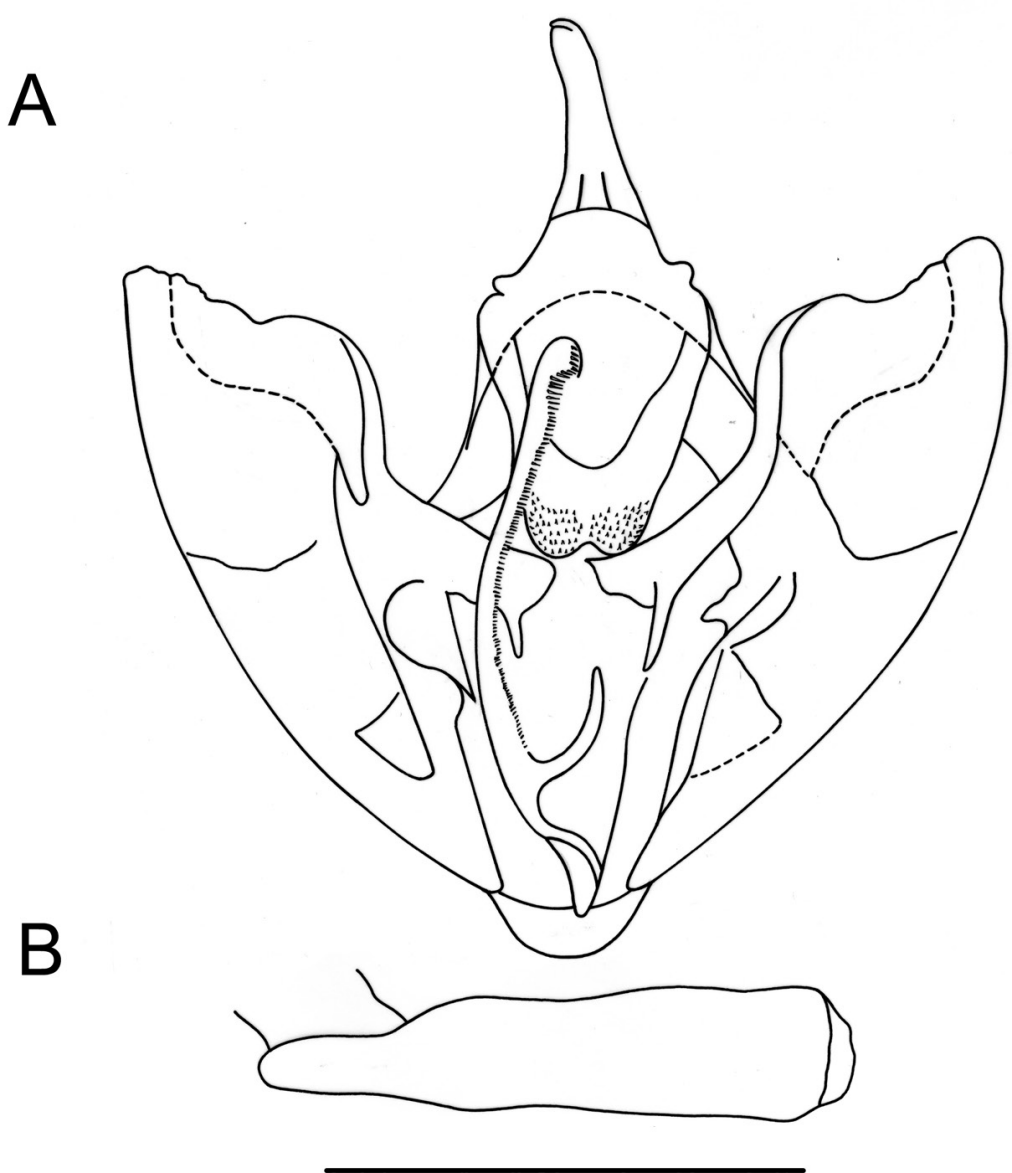
Genitalia of *Digonisgungnir* Ramos-González & Parra, sp. nov. (holotype) **A** male genitalia in ventral view **B** aedeagus in lateral view. Scale bar: 1 mm.

**Male** (Fig. 2M). Head: antennae slightly serrate; palpi long, one-third longer than the eye diameter, porrect; frons and vertex covered with juxtaposed whitish scales. Thorax: patagia covered with elongated whitish scales; tegulae covered with very pale yellowish piliform scales; tibial spur formula 0-2-4. Forewings: subtriangular with acute apex and outer margin excavated between apex and M_3_, with slight mucronate extension; fovea absent; background color straw-colored; antemedial band diffuse, marked by three light brown points at the level of radial, cubital, and anal veins respectively; medial band diffuse, light brown, slightly arched and more noticeable between the discal spot and the costa, zigzagging to the anal margin; postmedial band straight, diffuse, light brown, with five white points bordered in dark brown at the level of veins R_4_, R_5_, M_1_, M_2_, and M_3_, visible only on ventral surface; subterminal band diffuse and demarcated only by dark spots in subapical region, at the level of medial veins and on anal margin; discal spot visible, punctiform, and blackish. Hindwings: subrectangular with small mucronate apex at the level of M_3_; background color pale ashy; postmedial band light brown, slightly smoky, marked by elongated light brown spots at the level of veins, only visible on ventral surface; discal spot visible. Male genitalia (Fig. 6). Uncus conical, apex club-shaped; gnathos U-shaped with an expanded apex forming a pair of spinous lobes; valvae subrectangular, costa strongly sclerotized with a distal lobe before the apex, rounded, and cucullus extended beyond the apex of the costa; transtilla bifid; saccus subrounded; juxta poorly defined, pointed anteriorly, with a central depression, and with a furca curved to the left, long, surpassing the height of the transtilla, slightly arched spear-shaped, densely spiny dorsally, apex rounded; anellus sclerite weakly defined, only two subtriangular sclerites near the base of the furca are visible. Aedeagus tubular, straight; vesica without cornuti.

**Female.** Unknown.

##### Etymology.

The species name is a noun in apposition, referring to Odin’s spear (the chief god in Norse mythology), due to its longer and more armed furca within the genus. Gungnir is treated here as a neuter noun.

##### Distribution.

This species is found between the provinces of Elqui and Magallanes. It is distributed in parts of the biogeographic provinces of Coquimbo and Santiago, Central Chilean subregion; Maule, Valdivian Forest, and Magellanic Forest, Subantarctic subregion, Andean region.

##### Flight period.

Specimens were captured in December and February. There are no records for other months.

#### ﻿ Digonis apocrypha

Taxon classificationAnimaliaLepidopteraGeometridae

Ramos-González & Parra sp. nov.

988A3A15-C672-5EF7-970C-52D06A2AB89D

https://zoobank.org/458C9CE8-3466-4EEE-99A0-35F4E244557C

Figs 2N, O , 7

##### Type material.

***Holotype*.** Chile: 1 male; pinned; Ñuble, Las Trancas; 31-III-2011; G. Moreno leg.; “Holotype *Digonisapocrypha*” [red handwritten label], “UCCC_MZUC_Lep_0300” [Museum ID], “Mirg-021” [genitalia slide] (MZUC-UCCC). ***Paratypes*.** Chile — **Diguillín Prov.** • 1 male; pinned; Ñuble, Recinto; 06-II-2011; G. Moreno leg.; MZUC-UCCC • 1 male; pinned; same data as holotype but “UCCC_MZUC_Lep_0304” [Museum ID] (MZUC-UCCC) • 2 males; pinned; same data as holotype but 12-IV-2013; “UCCC_MZUC_Lep_1729” and “UCCC_MZUC_Lep_1730” [Museum ID]; MZUC-UCCC • 2 males; pinned; same data as holotype but 20-III-2011; “UCCC_MZUC_Lep_0330” and “UCCC_MZUC_Lep_0320” [Museum ID]; MZUC-UCCC • 1 male; pinned; same data as holotype but 16-IV-2010; “UCCC_MZUC_Lep_0303” [Museum ID]; MZUC-UCCC • 2 males; pinned; same data as holotype but 7-IV-2010; MZUC-UCCC • 1 male; pinned; same data as holotype but “UCCC_MZUC_Lep_0301” [Museum ID]; MZUC-UCCC • 1 male; pinned; same data as holotype but 29-V-2011; “UCCC_MZUC_Lep_0302” [Museum ID]; MZUC-UCCC • 1 male; pinned; same data as holotype but 18-III-2012; “UCCC_MZUC_Lep_1160” [Museum ID]; MZUC-UCCC. — **Valdivia Prov.** • 1 male; pinned; 09-IV-2010; Huilo-Huilo, Mocho-Choshuenco volcano; -39.911943; -71.969167; L. Roa and D. Vergara leg.; MZUC-UCCC. — **Capitán Prat Prov.** • 1 male; pinned; Cochrane; 11-IV-2008; Parra and Alvarado leg.; “sampled in scrub of Notro-Ñirre” [labeled] (*Embothriumcoccineum* (Proteaceae) - *Nothofagusantarctica* (Nothofagaceae)); MZUC-UCCC.

##### Diagnosis.

This species is distinguished from the other species in the genus by the presence of elongated blackish marks with small whitish point at the level of veins in the postmedial band of the forewings, which are not visible in ventral view. In the male genitalia, it differs from *D.aspersa* by the elongated and striated furca, more similar to that of *D.cervinaria*. It also differs from *D.cervinaria* because it has spines around the distal half of the furca. This species stands out for the presence of a U-shaped gnathos with an expanded apex like a denticulate plate, transtilla with a digitiform process at the apex, and aedeagus with a fine and elongated caecum, half the total length of the aedeagus.

##### Description.

**Figure 7.**
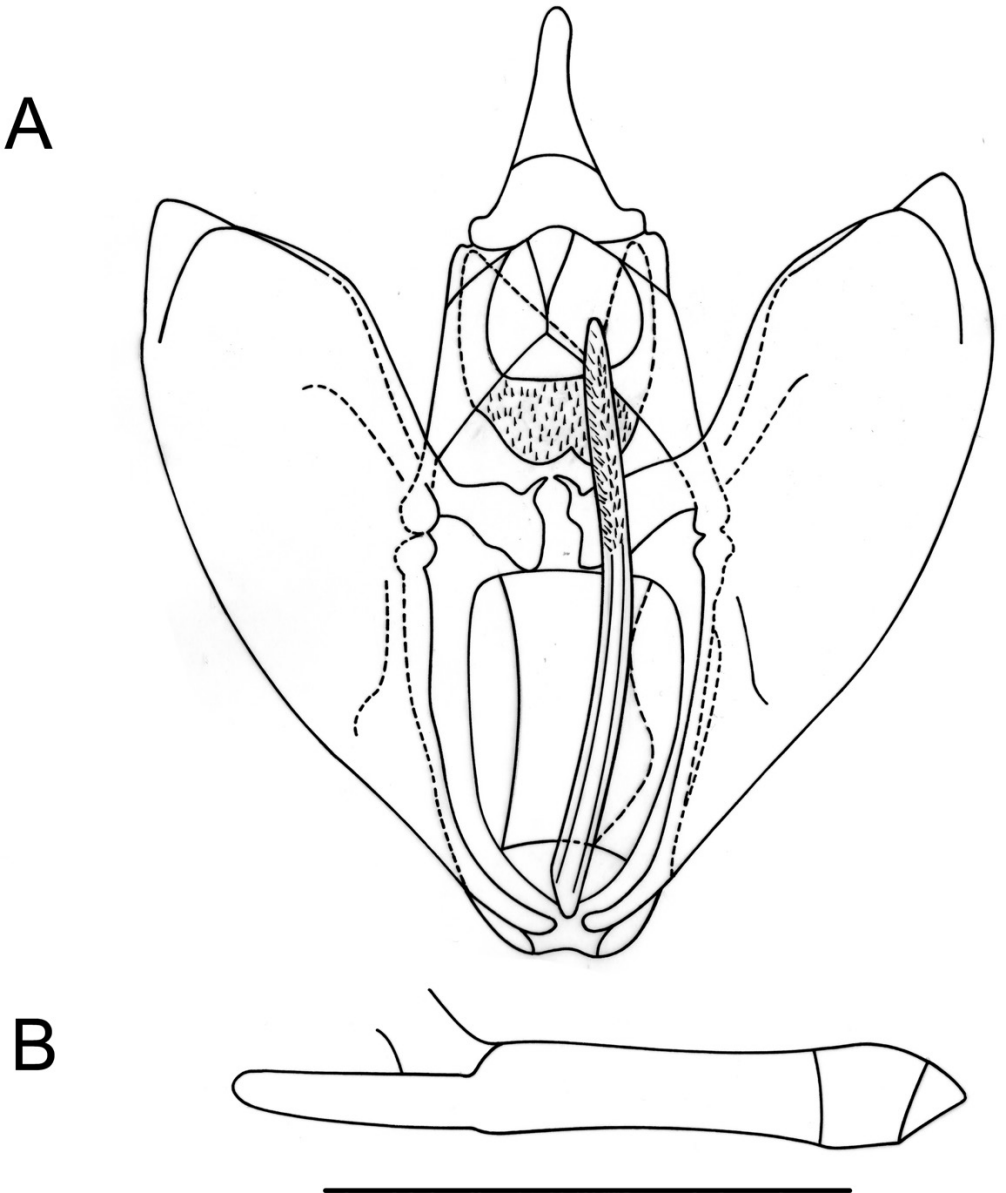
Genitalia of *Digonisapocrypha* Ramos-González & Parra, sp. nov. (holotype) **A** male genitalia in ventral view **B** aedeagus in lateral view. Scale bar: 1 mm.

**Male** (Fig. 2N, O). Head: antennae slightly serrate; palpi long, one-third longer than the eye diameter, porrect; frons and vertex covered with grayish brown scales. Thorax: patagia covered with elongated scales of same color as background; tegulae covered with piliform scales of same color as background; tibial spur formula 0-2-4. Forewings: subtriangular with acute apex and outer margin excavated between apex and M_3_, with slight mucronate extension; fovea absent; pale ashy background with a large number of small blackish spots scattered over the surface; antemedial band diffuse, slightly zigzagging, marked by three dark brown points at the level of radial, cubital, and anal veins respectively; medial band light brown, diffuse; postmedial band, diffuse, light brown, with bicolored spots (elongated blackish mark with the center marked by a whitish point) at the level of veins R_4_, R_5_, M_1_, M_2_, M_3_, CuA_1_, CuA_2_, and 1A+2A, not visible on ventral surface. Hindwings: subrectangular with small mucronate apex at the level of M_3_; pale ashy background, without visible bands or spots; discal spot visible. Male genitalia (Fig. 7). Uncus conical, apex club-shaped; gnathos U-shaped with an expanded apex like a denticulate plate; valvae subrectangular, costa strongly sclerotized with a distal lobe before the apex, rounded, and cucullus extended beyond the apex of the costa; transtilla bifid; saccus membranous, weakly defined; juxta poorly defined, with a central depression and furca, long, surpassing the height of the gnathos, club-shaped with two longitudinal stripes, densely spiny towards the distal half; anellus sclerite weakly defined. Aedeagus tubular, straight; fine, elongated caecum, half the total length of the aedeagus; vesica without cornuti.

**Female.** Unknown.

##### Etymology.

The species name is an adjective from Greek *aprocryphos* (“not genuine”), referring to the deceptive maculation pattern, superficially resembling *Digonisaspersa* Butler.

##### Distribution.

This species is found between the provinces of Diguillín and Capitán Prat. It is distributed in parts of the biogeographic provinces of Maule, Valdivian Forest, Magellanic Forest, Subantarctic subregion, in the Andean region.

##### Flight period.

Specimens were captured in February, March, April, and May. There are no records for other months.

#### ﻿ Phasmadigonis gen. nov.

Taxon classificationAnimaliaLepidopteraGeometridae

1C63ADF3-ED8E-568E-9310-FBD347F7CC6E

https://zoobank.org/F0922815-E334-4247-9CE0-9F98FD0DABE0

##### Type species.

*Digonisalba* Butler, 1882.

##### Diagnosis.

*Phasmadigonis* bears resemblance to *Digonodes* Warren, 1895, *Digonis* Butler, 1882, and *Gonogala* Butler, 1882, particularly due to the mucronate shape of the wings. However, *Phasmadigonis* can be distinguished by the presence of vein Sc connected by a vein to the single accessory cell in the forewings and by having vein Sc+R1 connected to radial trunk by a weak vein in the hindwings. *Phasmadigonis* is distinguished by the following genitalia characters: gnathos V-shaped with the absence of lobes or spines, spatulate transtilla, broad shovel-shaped juxta, furca armed with small sagittal spines and a dimple in the sclerite at its base, aedeagus with a digitiform apex, and vesica armed with a large spine.

##### Description.

**Figure 8.**
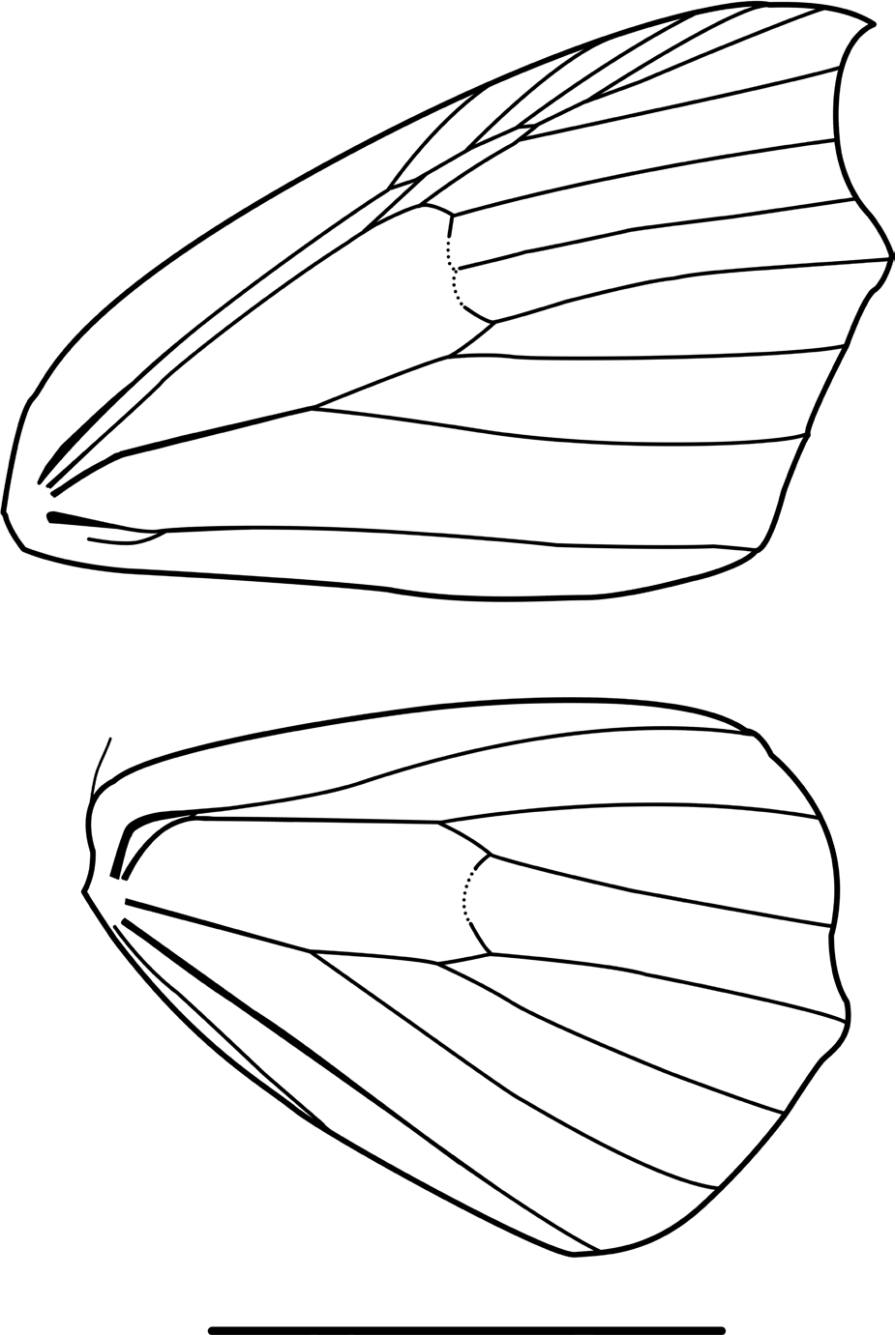
Wing venation of *Phasmadigonis* Ramos-González & Parra, gen. nov. Scale bar: 10 mm.

Antennae serrated in males and filiform in females. Thorax and abdomen with grayish scales. Forewings gray-lilac reticulated with white, lacking bands, subterminal region delimited only by a series of blackish spots at the level of R2, R3, R4, M1, M2, M3, CuA1, CuA2, and 1A+2A; costa margin in apical zone is slightly arched; outer margin is concave between apex and M3. Wing venation (Fig. 8): one accessory cell; Sc connected with accessory cell through a short vein, R1 and R2 arise from accessory cell, R3 and R4 are pedunculate, R5 terminates at termen; M2 equidistant from M1 and M3, M3 slightly arched and ending in small mucronate apex; CuA1 arises 1/10 before end of cell, CuA2 arises near middle of cell. Hindwings paler than forewings, with subterminal region delimited by series of dark spots at the level of veins. Sc+R1 connected to radial trunk by a weak transverse vein, Rs arises 1/6 before end of cell, M2 is absent. Male genitalia with rod-like uncus; gnathos V-shaped; valvae suboval; transtilla spatulate; furca spiny; vesica with prominent spine.

##### Etymology.

The generic name is formed by combining the Greek *phasma* (meaning phantom or apparition), with *Digonis* in reference to the false resemblance to moths of the genus *Digonis* Butler and the wing coloration; its gender is neuter.

##### Distribution.

Similar to its sole species, *P.alba* (Butler)

#### ﻿ Phasmadigonis alba

Taxon classificationAnimaliaLepidopteraGeometridae

(Butler, 1882) comb. nov.

4F3A3631-2702-54D5-A16E-695954A05455

Figs 2P , 9

Digonis alba Butler, 1882: 361; Bartlett-Calvert 1886: 333; Angulo and Casanueva 1981: 12; Scoble 1999: 229; Pitkin 2002: 248.

##### Material examined.

***Syntype*.** Chile • 1 female; pinned; Mountains of the hacienda of Cauquenes; T. Edmonds leg.; “Chili, 82-107” [labeled]; “Type” [labeled]; NHMUK.

##### Other material examined.

Chile — • 1 male; Chile; “Museo 5022” [Museum ID]; MNNC. — **Diguillín Prov.** • 1 male; Ñuble, Las Trancas; 18-III-2012; G. Moreno leg.; “UCCC-MUZC-Lep1173” [Museum ID]; “MMA1173” [genitalia slide]; MZUC-UCCC. — **Araucanía Region** • 1 male; Araucanía, II-1888, coll. n.n, “Museo 5029” [Museum ID]; MNNC; • 2 males; same data as for precedings but “Museo 5027” [Museum ID] and “Museo 5028” [Museum ID]; all MNNC. — **Chiloé Prov.** • 1 male; Mocopulli, Ruta 5 Sur km 1170, -42.368000, -73.728833, 182 m; 3-II-2017, leg. M. Ramos-G, M. Ramos-SM & C. Rose.

Argentina — • 1 male; Río Negro Prov., Bariloche; 29-I-1991; H. Ibarra-Vidal leg.; “HIV-0034” [Museum ID]; MHNC.

##### Additional records.

Chile. — **Talca Prov.** • Cipreses hydroelectric plant; -35.7867249, -70.8078157; 16-XII-2023; observed by César Picar and submitted to iNaturalist in: https://inaturalist.mma.gob.cl/observations/196336300. • Cipreses hydroelectric plant; -35.7865833685, -70.8078496903; 21-I-2024; observed by César Picar and submitted to iNaturalist in: https://inaturalist.mma.gob.cl/observations/197310545.

##### Diagnosis.

As for the genus.

##### Redescription.

**Figure 9.**
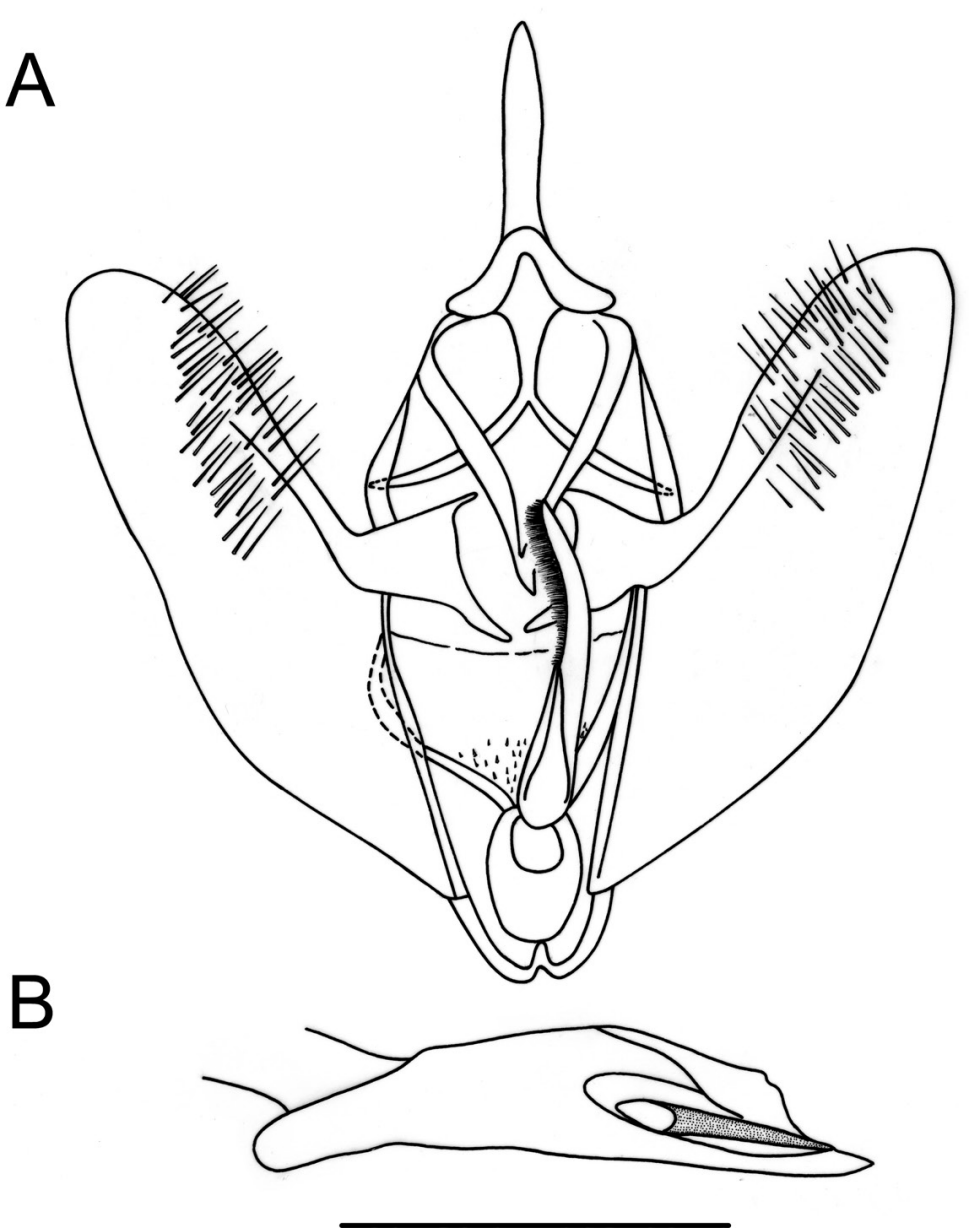
Genitalia of *Phasmadigonisalba*, comb. nov. **A** male genitalia in ventral view **B** aedeagus in lateral view. Scale bar: 1 mm.

**Male** (Fig. 2P). Head: antennae slightly serrated; short palpi, subequal to eye diameter, porrect; frons and vertex covered with juxtaposed brownish scales. Thorax: patagia covered with elongated scales of same color as background; tegulae covered with piliform scales of same color as background; tibial spur formula 0-2-4. Forewings: subtriangular with acute apex and outer margin excavated between apex and M_3_, with a slight mucronated extension; fovea absent; ground color gray-lilac reticulated with white; bands absent, only observable on wing surface are blackish points in subterminal area at the level of veins R_2_, R_3_, R_4_, M_1_, M_2_, M_3_, CuA_1_, CuA_2_, and 1A+2A, also visible on ventral side; discal spot visible, punctiform, and blackish. Hindwings: subrectangular with small mucronated apex at the level of M_3_; ground color ash-white, termen grayish; bands absent, only observable on wing surface is a row of blackish dots in subterminal area at the level of veins Sc+R_1_, Rs, M_1_, M_3_, CuA_1_, CuA_2_, and 1A; discal spot visible. Male genitalia (Fig. 9). Uncus straight, apex rod-like; gnathos V-shaped; valvae suboval, valvula and cucullus slightly setose, costa sclerotized; transtilla spatulate with sharp, projected vertices; saccus obcordate; juxta shovel-shaped, dorsally flattened and extended, with finger-shaped furca curved to the left, short, not surpassing the height of the transtilla,, densely spiny sagittally; anellus sclerite weakly defined. Aedeagus tubular, straight, apex digitiform; vesica armed with a large spine.

**Female.** Unknown.

##### Distribution.

This species is found between the provinces of Cachapoal and Chiloé (Chile) and Río Negro (Argentina). It is distributed in parts of the biogeographic provinces of Santiago, Central Chile subregion; Maule and Valdivian Forest, Subantarctic subregion, in the Andean region.

##### Flight period.

Specimens were captured in December, January, February, and March. There are no records for other months.

#### ﻿Tribe Nacophorini Forbes, 1948

##### ﻿ Gugnelve gen. nov.

Taxon classificationAnimaliaLepidopteraGeometridae

B09CE186-E521-5D94-866E-B94109AC2BDF

https://zoobank.org/5D5F1234-1907-4D23-A970-C051EF3D8DEA

###### Type species.

*Gugnelvebutleri* Ramos-González & Parra, sp. nov.

###### Diagnosis.

Externally, *Gugnelve* resembles *Euangerona* Butler and *Dectochilus* Warren. All three genera have an oblique curved band in the central area of the forewings. However, *Euangerona* and *Dectochilus* have a wavy termen, unlike *Gugnelve*, which has a smooth termen and a slightly falcate apex. The wing shape and antennae are similar to those of *Laninia* Orfila & Schajovskoy and *Macrolyrcea* Butler, but they differ significantly in various genital structures, such as the gnathos with a posteriorly directed apex and distally lobulated costa in *Laninia* and the spatulate uncus and wide oval valvae in *Macrolyrcea*. The V-shaped gnathos with a longitudinal row of spicules is reminiscent of *Euangerona* and *Dectochilus*, but in both of those genera, the anellus process is directed anteriorly, while in *Gugnelve*, it is oriented lateroposteriorly. The uncus and juxta resemble those of *Malleco* Rindge, but in *Gugnelve*, the uncus is glabrous, and the anellus process is trifid, while the gnathos of *Malleco* has multiple spicules on the lateral arms and apex. Finally, the new genus differs considerably in external characteristics and genitalia from other Andean Nacophorini like *Catophoenissa* Warren, 1894, *Catocalopsis* Rindge, 1971, *Talca* Rindge, 1971, and *Wichanraran* Parra, 2018. *Gugnelve* is distinguished by the following combination of genital characters: V-shaped gnathos with a row of 11 spicules at the apex, forked transtilla, halberd-shaped juxta laterally extended into a pair of tri-spined anellus processes, strongly sclerotized and glabrous, vesica armed with a series of cornuti, signum with a long blade-like ridge on a sclerotized patch.

###### Description.

**Figure 10.**
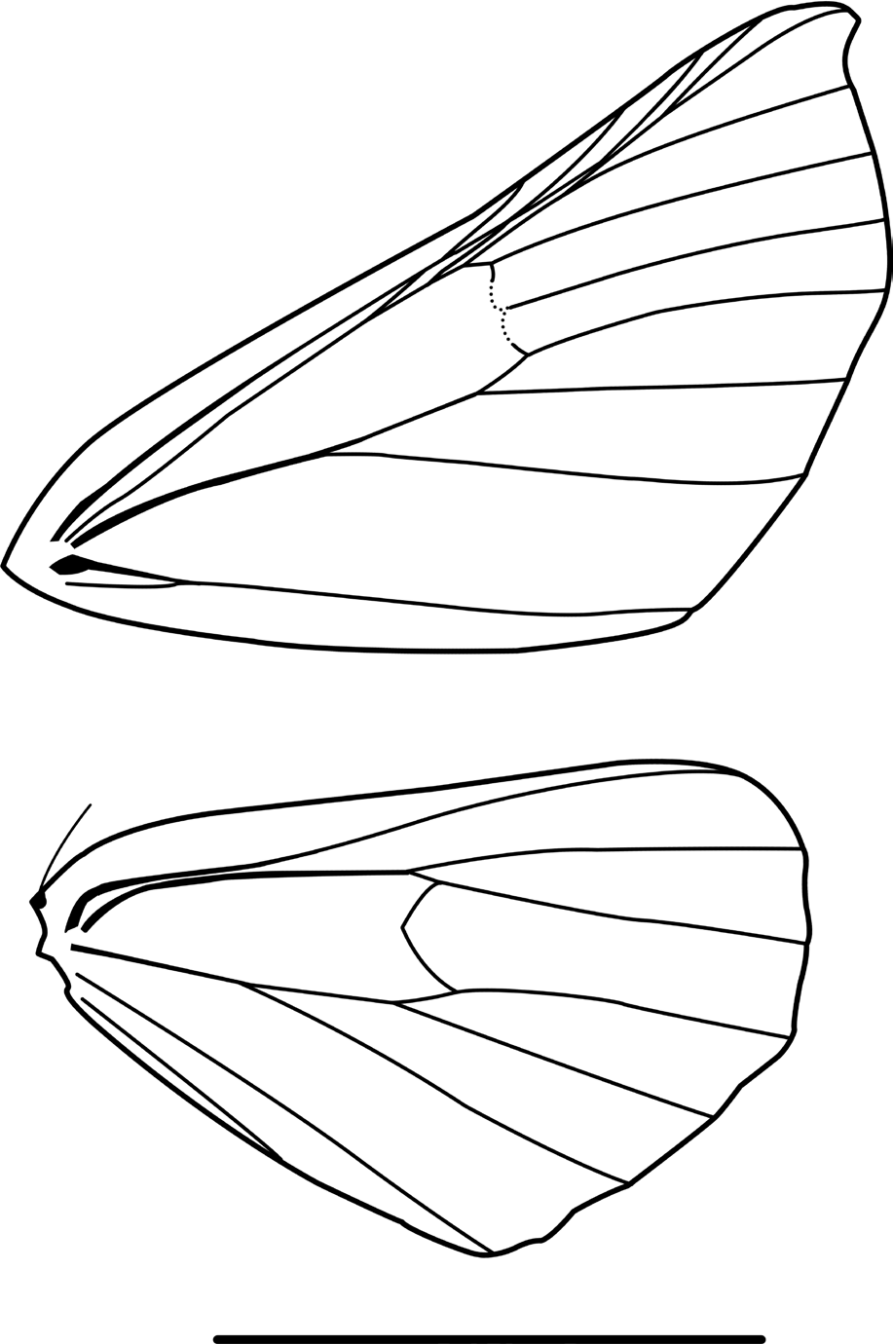
Wing venation of *Gugnelve* Ramos-González & Parra, gen. nov. Scale bar: 10 mm.

Antennae thickened and slightly serrated in males, filiform in females. Robust thorax with yellowish brown piliform scales. Abdomen with belt of white scales on A1 and A2. Forewings yellowish brown, subtriangular, slightly falcate, with sinuous and inclined antemedial and postmedial bands. Wing venation (Fig. 10). Two accessory cells, second twice as long as first; Sc in contact with first accessory cell, R1 originates from middle of second accessory cell, R2 and R3+4 connate, R3 and R4 pedunculate, R5 terminates at termen; M2 equidistant from M1 and M3, M3 slightly arched; CuA1 originates 1/8 before end of cell, CuA2 originates near middle of cell. Hindwings paler than forewings, with yellowish brown piliform scales on the termen. Sc+R1 in contact with radial trunk up to middle of cell, Rs originates 1/12 before end of cell, M2 absent. Male genitalia with V-shaped gnathos, expanded apex with 11 small variably-sized denticles towards the center; valvae sub-rhomboidal; juxta sub-halberd-shaped, laterally extended into a pair of tri-spined, strongly sclerotized processes. Female genitalia with signum with long blade-like ridge on sclerotized patch.

###### Etymology.

The generic name is derived from the Mapudungun language (spoken by the Mapuche, the largest indigenous group in Chile), wüṉyelfe, meaning bright star or Venus, in reference to its yellowish coloration; its gender is neuter.

###### Distribution.

As for its only species, *G.butleri* Ramos-González & Parra, sp. nov.

##### ﻿ Gugnelve butleri

Taxon classificationAnimaliaLepidopteraGeometridae

Ramos-González & Parra sp. nov.

0B8306E5-F889-5157-A825-1007D764CD71

https://zoobank.org/80882365-C942-46DB-AAD3-FC68E5CE32D8

Figs 2Q, R , 11

Erosina cervinaria (Blanchard, 1852); Butler 1882: 347 pl. 16, fig. 4; Bartlett-Calvert 1886: 331; Angulo and Casanueva 1981: 12 [misidentification of Ennomoscervinaria Blanchard].

###### Type material.

***Holotype*.** Chile • 1 male; Cordillera Prov., Santiago, Guayacan; XII-1950; “Holotype *Gugnelvebutleri*” [red handwritten label], “Mirg-017” [genitalia slide] (MZUC-UCCC). ***Allotype*.** Chile • 1 female, Cordillera Prov., Santiago, Guayacan; I-1951; “Allotype *Gugnelvebutleri*” [red handwritten label], “Mirg-018” [genitalia slide] (MZUC-UCCC). ***Paratypes*.** Chile — **Limarí Prov.** • 2 males, Ovalle, Quebrada Seca; 28/29-XI-1997; ZSM. — **Cordillera Prov.** • 4 females and 1 male, same data as Holotype; • 1 female and 1 male, same data as allotype.

###### Other material examined.

Chile — **Cachapoal Prov.** • 1 female; Termas de Cauquenes; 11-I-1953; “Museo 4516” [Museum ID]; MNNC.

###### Additional records.

Chile. — **Cachapoal Prov.** • Mountains of the hacienda of Cauquenes; January; T. Edmonds leg.; NHMUK (Butler, 1882) — **Talca Prov.** • Parque Natural Tricahue; -35.70869903, -71.08727243; 5-I-2020; observed by Vicente Pantoja and submitted to iNaturalist in: https://inaturalist.mma.gob.cl/observations/37300513. • Cipreses hydroelectric plant; -35.8099272997, -70.8359745; 17-XII-2022; observed by César Picar and submitted to iNaturalist in: https://iNaturalist.mma.gob.cl/observations/144651054. • Cipreses hydroelectric plant; -35.8096577, -70.8353316; 10-I-2024; observed by César Picar and submitted to iNaturalist in: https://inaturalist.mma.gob.cl/observations/196337433.

###### Diagnosis.

As for the genus.

###### Description.

**Figure 11.**
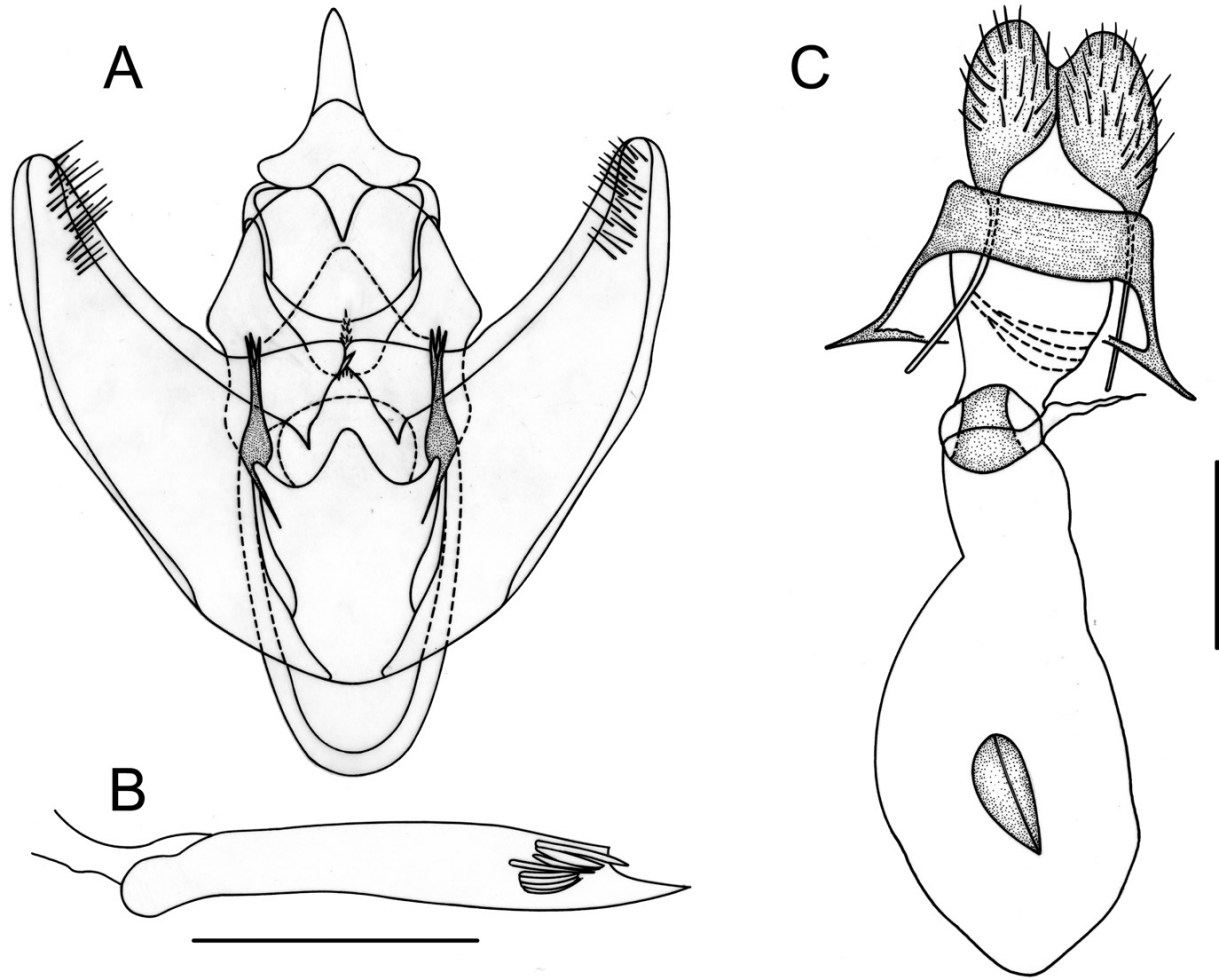
Genitalia of *Gugnelvebutleri* Ramos-González & Parra, sp. nov. (holotype and allotype) **A** male genitalia in ventral view **B** aedeagus in lateral view **C** female genitalia in ventral view. Scale bars: 1 mm.

**Male** (Fig. 2Q). Head: antennae slightly serrated; palpi short, subequal to the eye diameter, porrect; frons and vertex covered with juxtaposed brownish scales. Thorax: Patagia covered with elongated scales of same color as background; tegulae covered with piliform scales of same color as background; tibial spur formula 0-2-4. Forewings: slightly falcate, subtriangular; fovea absent; background color yellowish brown; antemedial band dark yellowish brown, externally bordered by pale brown, oblique, extending toward the medial area, near the end of the discal cell where it curves toward the antemedial sector to the costa, forming an acute arc; postmedial band dark yellowish brown, externally bordered by pale brown, slightly sinuous, oblique, and slightly curved, extending to the subapical sector; discal spot not visible. Hindwings: suboval; background color pale brown without bands; discal spot not visible. Male genitalia (Fig. 11A, B). Uncus conical; socii slightly setose; gnathos V-shaped with sinuous arms, expanded apex with 11 small denticles of varying sizes toward the center; valvae sub-rhomboidal, costa and anterior margin sclerotized; transtilla fork-shaped; saccus suboval; juxta sub-halberd-shaped, laterally extended into a pair of tri-spined strongly sclerotized processes; anellus sclerite weakly defined. Tubular aedeagus, straight; vesica armed with cornuti, formed by 11 grouped spines of different sizes.

**Female** (Fig. 2R). Similar to male but with simple filiform antennae, dark yellowish brown falcate forewings, and slightly crenulated pale brownish gray subrectangular hindwings. Female genitalia (Fig. 11C). Ductus bursae half the length of the corpus bursae; antrum funnel-like; corpus bursae membranous, subpyriform, signum with a long blade-like ridge on a sclerotized patch; posterior apophyses three times longer than anterior apophyses.

###### Etymology.

The specific name is dedicated to Arthur Gardiner Butler, an ornithologist, arachnologist, and entomologist of the 19^th^ century, for his contribution to the study of Lepidoptera in Chile.

###### Distribution.

This species is found between the provinces of Limarí and Talca. It is distributed in parts of the biogeographic provinces of Coquimbo and Santiago, Central Chilean subregion; Maule, Subantarctic subregion, in the Andean region.

###### Flight period.

Specimens were captured in November, December, and January. There are no records for other months.

## ﻿Discussion

The genus *Digonis*, described by Butler in 1882, initially included *D.aspersa*, *D.cuprea*, *D.punctifera*, and *D.alba*. Additionally, Butler (1882) described various varieties for *cuprea* and *punctifera*, differentiating them based on the coloration of the forewings. However, the analysis of the genital structures of *D.punctifera* indicates that these differences in coloration are not sufficient to separate them into distinct species or subspecies within the genus, which is why they are synonymized.

The inclusion of the species *D.gungnir* Ramos-González & Parra, sp. nov. and *D.apocrypha* Ramos-González & Parra, sp. nov. in the genus *Digonis* is justified by the congruence in their external characteristics and genitalia. The distinction from their sister species is primarily focused on male genitalia characteristics. Therefore, the genus *Digonis* is composed of the following five species: *D.aspersa*, *D.cervinaria*, *D.punctifera*, *D.gungnir* Ramos-González & Parra, sp. nov., and *D.apocrypha* Ramos-González & Parra, sp. nov. Its validity as a taxon within the tribe Ennomini is supported by the presence of a membranous and weakly developed saccus, valvae with well-sclerotized outstanding costa, and well-developed furca (Beljaev 2008).

Based on the analysis of genital structures and wing venation, a strong incongruence is recognized between the species *D.alba* Butler and the rest of the species in *Digonis*. This aligns with what Pitkin (2002) indicated, suggesting that *D.alba* should be excluded from the genus due to certain incongruences with genital characters. The main differences between both genera lie in the shape of the valvae, furca, and wing venation configuration, a combination of characters not present in any other Ennomini genus in the Andean region (e.g., *Syncirsodes* Butler, *Gonogala* Butler, *Microclysia* Butler, *Perusia* Herrich-Schäffer, *Eusarca* Hübner), which supports the proposal of *Phasmadigonis* as a new genus. Additionally, we report the first record of this taxon for Argentina, specifically for the province of Río Negro.

The different species of the genus *Digonis* have distributions that cover most of the vegetation formations (sensu Luebert and Pliscoff 2018) in central-southern Chile. However, the highest species richness appears to be associated with the high-Andean deciduous forest of the Cordillera de Chillán (sensu Gajardo 1994). The lack of records in other areas may be due to a lack of comprehensive sampling, particularly in the central-northern zone of Chile (north of Santiago). Future studies should address the rearing of different species to describe their host plants and immature stages, currently unknown.

Blanchard (1852) described the species *Ennomoscervinaria* based on a specimen from Coquimbo, providing a brief and imprecise description, and without including figures. Later, Guenée (1858) redescribed it in more detail, characterizing it by the elongated whitish marks on the wing veins, visible in both dorsal and ventral views. Furthermore, Guenée proposed a new name for this species, arguing that “*cervinaria*” was already used for a species of *Eubolia* Duponchel, 1829 (current *Larentiaclavaria* Duponchel, 1845). Subsequently, Butler (1882) incorporated this species into the genus *Erosina* Guenée, 1858, arguing that, if his determination of the specimen used is correct, it has a second oblique but indistinct angular line from the costa to the inner margin, almost parallel to the line described by Blanchard (1852) in his description. Additionally, Butler (1882) added a sketch of the species for easier future determination. Finally, Scoble (1999) included it in the genus *Digonis*. However, the analysis of the type material reveals that the specimens determined as *Ennomoscervinaria* by Butler (1882) and subsequent authors are not congruent with the holotype of *Ennomoscervinaria* Blanchard. The external characteristics and genitalia of *Ennomoscervinaria* correspond to those of *Digoniscuprea* and its varieties, so these are synonymized.

The analysis of specimens incorrectly determined as *E.cervinaria* by Butler reflects a morphological correspondence with the concept of “Nacophorini” (sensu Rindge 1973, 1983), which brought together the Ennominae with a broad and “hairy” thorax that now form parts of the tribes Euangeronini, Odontoperini, and Nacophorini in part (Brehm et al. 2019). The most characteristic feature in the male genitalia corresponds to the row of spicules in the sagittal plane of the gnathos and the trifid, strongly sclerotized posterolaterally directed process of the anellus. This form and configuration of the gnathos are present in the genera *Euangerona* Butler and *Dectochilus* Butler; however, the process of the anellus is not congruent with both genera or any other Euangeronini, which show a process of the anellus pointing anteriorly and a highly developed setose socius (possible synapomorphies of the tribe). On the other hand, it shares with *Mallomus* Blanchard the spine-shaped form of the anellus process; however, in *Mallomus*, it appears as a single sclerotized spine, and the valvae have various modifications absent in *Gugnelve* gen. nov. such as a membranous harpe and digitiform processes on the cucullus. It was also not possible to assign this species to any of the Nacophorini genera in southern South America treated by Rindge (1973, 1983), Pitkin (2002), Parra and Pascual-Toca (2003), or Parra (2018). For this reason, *Gugnelvebutleri* Ramos-González & Parra, gen. et sp. nov. is provisionally included in the Nacophorini.

Finally, this revision will serve as the basis for future molecular phylogenetic analyses that will help clarify the evolutionary relationships among species and related taxa. With this work, the number of Ennomini species present in Chile increases to 36 (Parra et al. 2009; Bocaz et al. 2016; Brehm et al. 2019), and the Nacophorini to 20 (Pitkin 2002; Brehm et al. 2019). These numbers are likely to increase in the future, considering that there have been few taxonomic revisions of Ennominae in the Andean region so far.

## Supplementary Material

XML Treatment for Digonis

XML Treatment for Digonis aspersa

XML Treatment for Digonis cervinaria

XML Treatment for Digonis punctifera

XML Treatment for Digonis gungnir

XML Treatment for Digonis apocrypha

XML Treatment for Phasmadigonis

XML Treatment for Phasmadigonis alba

XML Treatment for Gugnelve

XML Treatment for Gugnelve butleri

## ﻿Additional information

### Author ORCIDs

Mario I. Ramos-González https://orcid.org/0000-0002-5385-6836

Carlos Zamora-Manzur https://orcid.org/0000-0001-7755-4077

Luis E. Parra https://orcid.org/0000-0002-3271-0037

### Data availability

All of the data that support the findings of this study are available in the main text.
